# Supplementary material for: Advanced neural activity mapping in brain organoids via field potential imaging with ultra-high-density CMOS microelectrode arrays
Source: Front Neurosci. 2025 Aug 13;19:1634582. doi: 10.3389/fnins.2025.1634582 (PMC12392918; doi:10.3389/fnins.2025.1634582)
Supplement: Supplementary file 1 [file Table_1.docx]

Supplementary Material

**Supplementary Table 1.** Composition of culture media used for cerebral organoid generation, prepared according to the STEMdiff™ Cerebral Organoid Kit protocol (08570, STEMCELL Technologies), including Y-27632 as specified in the manufacturer's instructions.

| **Medium** | **Component** | **Source** | **Cat. No.** | **Amount** |
| --- | --- | --- | --- | --- |
| EB seeding medium | STEMdiff Cerebral Organoid Supplement A | STEMCELL Technologies | 08574 | 10 mL |
|  | STEMdiff Cerebral Organoid Basal Medium 1 | STEMCELL Technologies | 08572 | 40 mL |
|  | Y-27632 (10mM) | FUJIFILM Wako | 036-24023 | 0.05 mL |
| EB Formation Medium | STEMdiff Cerebral Organoid Supplement A | STEMCELL Technologies | 08574 | 10 mL |
|  | STEMdiff Cerebral Organoid Basal Medium 1 | STEMCELL Technologies | 08572 | 40 mL |
| Induction Medium | STEMdiff Cerebral Organoid Supplement B | STEMCELL Technologies | 08575 | 0.5 mL |
|  | STEMdiff Cerebral Organoid Basal Medium 1 | STEMCELL Technologies | 08572 | 49.5 mL |
| Expansion Medium | STEMdiff Cerebral Organoid Supplement C | STEMCELL Technologies | 08576 | 0.25 mL |
|  | STEMdiff Cerebral Organoid Supplement D | STEMCELL Technologies | 08577 | 0.5 mL |
|  | STEMdiff Cerebral Organoid Basal Medium 2 | STEMCELL Technologies | 08573 | 24.25 mL |
| Maturation Medium | STEMdiff Cerebral Organoid Supplement E | STEMCELL Technologies | 08578 | 4.5 mL |
|  | STEMdiff Cerebral Organoid Basal Medium 2 | STEMCELL Technologies | 08573 | 224.5 mL |

**Supplementary Table 2.** Composition of culture media for midbrain–striatal assembloid generation using STEMdiff™ organoid kits (100-1096 and 08620, STEMCELL Technologies), including Y-27632 and region-specific supplements recommended by the manufacturer.

| **Medium** | **Component** | **Source** | **Cat. No.** | **Amount** | |
| --- | --- | --- | --- | --- | --- |
| Organoid Formation Medium | STEMdiff™ Neural Organoid Basal Medium 1 | STEMCELL Technologies | 08621 | 20 mL | |
| Midbrain Organoid Expansion Medium | STEMdiff™ Neural Organoid Basal Medium 2 | STEMCELL Technologies | 08622 | 49 mL | |
|  | STEMdiff™ Neural Organoid Supplement A | STEMCELL Technologies | 08623 | 1 mL | |
|  | STEMdiff™ Neural Organoid Supplement K | STEMCELL Technologies | 100-1094 | 0.1 mL | |
|  | STEMdiff™ Neural Organoid Supplement L | STEMCELL Technologies | 100-1095 | 0.1 mL | |
| Striatal Organoid Expansion Medium | STEMdiff™ Neural Organoid Basal Medium 2 | STEMCELL Technologies | 08622 | 49 mL | |
|  | STEMdiff™ Neural Organoid Supplement A | STEMCELL Technologies | 08623 | 1 mL | |
|  | Actin A (10 µg/ml) | PeproTech | 120-14P | 0.25 mL | |
|  | IWP-2 (2.5 mM) | Selleck Chemicals | S7085 | 0.05 mL | |
|  | SR11237 (100 µM) | Sigma Aldrich | S8951 | 0.05 mL | |
| Organoid Differentiation Medium | STEMdiff™ Neural Organoid Basal Medium 2 | STEMCELL Technologies | 08622 | 49 mL | |
|  | STEMdiff™ Neural Organoid Supplement A | STEMCELL Technologies | 08623 | 1 mL |  |
|  | STEMdiff™ Neural Organoid Supplement C | STEMCELL Technologies | 08625 | 0.05 mL |  |
| Organoid Maintenance Medium | STEMdiff™ Neural Organoid Basal Medium 2 | STEMCELL Technologies | 08622 | 49 mL |  |
|  | STEMdiff™ Neural Organoid Supplement A | STEMCELL Technologies | 08623 | 1 mL |  |

**Supplementary Table 3.** Summary of statistical results for pharmacological assays. This table presents the detailed outcomes of statistical analyses performed for each pharmacological condition, including p-values from one-way ANOVA and Dunnett’s post hoc tests, along with effect size estimates. Eta squared (η²) values indicate the overall treatment effect across drug concentrations relative to baseline, and Cohen’s d values quantify the magnitude of pairwise differences between baseline and each concentration.

| **Figure No.** | **Comparison** | **Test Used** | **n** | **Test Statistic** | **p-value** | **Effect Size** | **Effect Size Type** |
| --- | --- | --- | --- | --- | --- | --- | --- |
| Fig. 1D | All groups | One-way ANOVA | 3/group | F (3,16) = 4.55 | 0.0384 | 0.63059 | Eta squared |
|  | Before vs. L-DOPA 0.3 µM | Dunnett post-hoc (vs. Before) | 3 | — | 0.2079 | 0.86681 | Cohen’s d |
|  | Before vs. L-DOPA 3 µM | Dunnett post-hoc (vs. Before) | 3 | — | 0.1452 | 2.60 | Cohen’s d |
|  | Before vs. L-DOPA 30 µM | Dunnett post-hoc (vs. Before) | 3 | — | 0.0158 | 5.4641 | Cohen’s d |
| Fig. 2D | All groups | One-way ANOVA | 404/group | F (3,1612) = 69.3 | 2.00E-16 | 0.11428 | Eta squared |
|  | Before vs. L-DOPA 0.3 µM | Dunnett post-hoc (vs. Before) | 404 | — | 0.904 | 0.032857 | Cohen’s d |
|  | Before vs. L-DOPA 3 µM | Dunnett post-hoc (vs. Before) | 404 | — | 0.00001 | 0.62974 | Cohen’s d |
|  | Before vs. L-DOPA 30 µM | Dunnett post-hoc (vs. Before) | 404 | — | 0.00001 | 0.66074 | Cohen’s d |
| Fig. 3C | All groups | One-way ANOVA | 3/group | F (3,8) = 9.25 | 0.00558 | 0.776 | Eta squared |
|  | Before vs. picrotoxin 0.1 µM | Dunnett post-hoc (vs. Before) | 3 | — | 0.00477 | 3.13 | Cohen’s d |
|  | Before vs. picrotoxin 1 µM | Dunnett post-hoc (vs. Before) | 3 | — | 0.0279 | 2.35 | Cohen’s d |
|  | Before vs. picrotoxin 10 µM | Dunnett post-hoc (vs. Before) | 3 | — | 0.00482 | 3.27 | Cohen’s d |
| Fig. 3E | All groups | One-way ANOVA | 3/group | F (3,8) = 8.51 | 0.00718 | 0.761 | Eta squared |
|  | Before vs. MK-801 0.1 µM | Dunnett post-hoc (vs. Before) | 3 | — | 0.994 | -0.125 | Cohen’s d |
|  | Before vs. MK-801 1 µM | Dunnett post-hoc (vs. Before) | 3 | — | 0.0501 | -3.21 | Cohen’s d |
|  | Before vs. MK-801 10 µM | Dunnett post-hoc (vs. Before) | 3 | — | 0.00713 | -2.65 | Cohen’s d |
